# Long-term effects of mepolizumab in patients with severe eosinophilic asthma: a 6-year real-life experience

**DOI:** 10.3389/fphar.2024.1449220

**Published:** 2024-08-08

**Authors:** Anna Agnese Stanziola, Claudio Candia, Gerardo Nazzaro, Antonio Caso, Claudia Merola, Lorena Gallotti, Mauro Maniscalco

**Affiliations:** ^1^ Department of Clinical Medicine and Surgery, University of Naples “Federico II”, Naples, Italy; ^2^ Istituti Clinici Scientifici Maugeri IRCCS, Pulmonary Rehabilitation Unit of Telese Terme, Telese Terme, Italy; ^3^ Department of Respiratory Medicine, Azienda Ospedaliera dei Colli, Naples, Italy

**Keywords:** severe asthma, mepolizumab, biomarkers, outcome, disability, rehabilitation, occupational medicine

## Abstract

**Background:**

Severe eosinophilic asthma (SEA) is often linked to a dysregulation in the Interleukin-(IL)-5 axis. Mepolizumab, a humanized monoclonal antibody, reduces eosinophils by directly binging to IL-5, potentially restoring homeostatic eosinophil biology, with a significant impact on quality of life, acute exacerbations and oral corticosteroids (OCS) elimination in SEA patients. While its short- and middle-term effects are well described, no study has so far investigated its long-lasting effects in SEA patients. The aim of our study was therefore to explore the effects of a long-term, six-year continuous treatment with mepolizumab on clinical control and clinical remission in a cohort of SEA patients.

**Methods:**

We conducted a retrospective review of clinical records of patients who were prescribed mepolizumab between June 2017 and April 2018. We collected demographical, functional, and clinical data from visits performed at baseline and then at the specified timepoints and checked if patients had reached clinical remission after 6 years. We assessed asthma control test (ACT), exacerbation rate, and OCS elimination dose at 6 years. Clinical Remission (CR) was defined on the basis of the elimination of OCS and the contemporary presence of all the following: 1) stable lung function; 2) no exacerbation in the previous 12 months; 3) acceptable symptom control (ACT ≥ 20).

**Results:**

Of 86 patients screened, 62 were included in the final analysis. Our study suggests that mepolizumab is effective and well tolerated after a six-year course of continuous treatment in patients with SEA. We reported a prevalence of 28 (46.8%) patients who reached complete CR at 72 months from the treatment start. 75% of patients eliminated the maintenance OCS already after 1 year of treatment; this proportion reached the 87% within the sixth year of treatment.

**Conclusion:**

Mepolizumab proved to be effective in real-life after 6 years of treatment, inducing a complete clinical remission in the 46.8% of patients, with sustained improvements in quality of life, exacerbation rate, OCS intake and lung function.

## 1 Introduction

Severe Asthma (SA) is a chronic condition characterized by the lack of symptom control despite an optimal compliance to maximal inhalation therapy (Global Initiative for Asthma, 2023). SA is also characterized by a higher risk of experiencing severe acute exacerbations with subsequent hospitalization (McDonald and Gibson, 2012), as well as an increased need for oral corticosteroids (OCS) to achieve symptom control. Within the realm of SA, severe eosinophilic asthma (SEA) (Nelson et al., 2020) is the most common phenotype. The pathogenesis of SEA is related to a dysregulation of the eosinophils (Alvarez Puebla et al., 2018), whose survival and proliferation are mostly dependent on Interleukin (IL)-5, a key mediator in the type 2 (T2) inflammation (Sanderson, 1992). In this clinical setting, the relatively recent discovery of specific anti-IL5 biological drugs has brought unprecedented changes in the management of SEA patients. Among such drugs, mepolizumab, a humanized monoclonal antibody, has proven able to improve asthma control, reduce the annual exacerbation rate (AER) and the use of maintenance OCS, as well as their related consequences (Pelaia et al., 2017), with evidence deriving from both randomized-controlled clinical trials (RCTs) and real-life observational studies (Domingo Ribas et al., 2021; Maglio et al., 2021; Pavord et al., 2022). However, only few studies (Khatri et al., 2019; Khurana et al., 2019; Domingo Ribas et al., 2021; Moore et al., 2022; Pilette et al., 2022; Fyles et al., 2023; Strauss et al., 2023) evaluated the efficacy and safety of mepolizumab for up to 3 years of continuous treatment, confirming its positive impact on quality of life, lung function, chronic use of OCS and exacerbation rate. Unfortunately, a 3-year evaluation period may not be deemed appropriate to evaluate long term aspects such as: 1) loss of efficacy, 2) predictors of enhanced or reduced response to treatment, and 3) correct timing of treatment initiation. Moreover, in recent years the novel concept of clinical remission (CR) and its predictor factors has intrigued clinicians and researchers. In particular, a *post hoc* analysis of the REDES study (Pavord et al., 2023) identified a subgroup of “super-responders” to mepolizumab, with variable proportions on the basis of the criteria proposed by Menzies-Gow (Menzies-Gow et al., 2020) involving three or four factors. However, although several criteria for the definition of CR have been proposed so far, including lung function, fractional exhaled nitric oxide (FeNO) levels, bronchodilator reversibility, bronchodilators and OCS use, and AER, a universal consent has not been reached (Thomas et al., 2022). In this context, the definition of CR proposed by Canonica et al. (2023), who established the presence of clinical remission on the basis of OCS elimination, absence of acute exacerbations, improvements in asthma control and stabilization of lung function, appears to be the most comprehensive and practical for routine clinical application. In the present single-center, retrospective, observational cohort study we therefore aimed to investigate the real-life long-term effectiveness of mepolizumab on clinical control including improvement of asthma control test, reduction of AER and maintenance OCS dose reduction or elimination in patients with SEA during 6 years of continuous treatment. The number of patients who reached CR as proposed by Canonica et al. (2023), the variations in both lung function and expression of inflammatory markers, and the presence of adverse drug reactions (ADRs) were also assessed.

## 2 Materials and methods

### 2.1 Patients and study design

Patients followed at our outpatient clinic for severe asthma (Azienda Ospedaliera dei Colli–Presidio Ospedaliero “V. Monaldi,” Naples, Italy) were screened for inclusion.

The inclusion criteria included: age ≥ 18 years, clinical diagnosis of SEA, according to the most updated guidelines (Chung et al., 2014), naïve to previous biological treatment for SEA, eligibility for treatment with mepolizumab, according to the indications of the Italian Regulatory Agency (AIFA) (Italian Medicines Agency) (Supplementary Table S1), without any discontinuation in the previous 6 years. Patients were excluded if they discontinued the administration of mepolizumab for any reason, if they did not comply with the inhalatory therapy, if they had concomitant malignancies, severe cardiovascular diseases, if they did not consent to the participation in the study, and finally if their records were burdened by a several amount of missing key data.

Data were collected from clinical records corresponding to visits performed at baseline, before the administration of mepolizumab (T_0_), and usually after 6 months (T_6_), 1 year (T_12_) and each year afterwards (T_24_-T_72_) up to 6 years. T_0_, T_12_ and T_72_ were considered as the main timepoints for the analysis.

Whenever feasible, the study was conducted according to the STROBE guidelines for observational studies (von Elm et al., 2007). The study protocol was compliant with the Declaration of Helsinki and was reviewed by the Institutional Review Board Campania 2 (approval number AOC/0019020/2024). We made the effort of contacting all patients involved and obtain written informed consent.

### 2.2 Study procedures

All patients included in the current study underwent the procedures described below during the observational period, and at least at the most important timepoints, namely baseline (T_0_), 1 year (12 months) after baseline (T_12_), and 6 years (72 months) after baseline (T_72_).

Functional parameters were evaluated through a spirometry conducted at each time interval and were reported according to the most recent guidelines from the American Thoracic Society/European Respiratory Society (ATS/ERS) (Stanojevic et al., 2022). The exam was performed using a Master Screen Body^®^ (Jaeger CareFusion, Yorba Linda, CA, United States). Pulmonary function test results, including forced vital capacity (FVC), forced expiratory volume in the first second (FEV_1_), and FEV_1_/FVC ratio, were recorded both as absolute values and as a percentage of theoretical values (FEV_1_%, FVC%). Peripheral venous blood analysis was conducted at each timepoint in order to evaluate the blood eosinophil count (BEC). Fractional exhaled nitric oxide (FeNO) levels were assessed using a portable device (Hyp’AirFeNO; MediSoft, Sorinnes, Belgium) as previously described (Molino et al., 2019). A positive test result was considered whenever values were higher or equal to 25 ppb, in accordance with the latest ATS guidelines (Dweik et al., 2011). The maintenance OCS dose and AER were assessed from documentations and medical examinations exhibited by patients. At each timepoint, asthma control was assessed through a validated questionnaire, the Asthma Control Test, ACT (Nathan et al., 2004).

Following the proposal by Canonica et al. (2023) we defined complete clinical remission (cCR) as the elimination of daily OCS intake and the contemporary presence of all the following: 1) stable lung function; 2) no exacerbation in the previous 12 months; 3) acceptable symptom control (ACT ≥ 20). Partial clinical remission (pCR) was instead defined as the elimination of daily OCS intake and the coexistence of at least two of the aforementioned criteria. The presence of cCR and pCR was assessed at 12 and at 72 months.

Finally, we assessed the prevalence and characteristics of ADRs registered during the observational time period.

### 2.3 Statistical analysis

Statistical analysis was performed with SPSS version 29.0 (IBM, Chicago, Illinois, United States). Continuous variables were expressed as mean ± standard deviation (SD) or as median (interquartile range, IQR) according to the presence of a normal or skewed distribution. Such variables were then compared with a paired Student’s t-test or a Wilcoxon Rank Test, accordingly. Categorical variables were reported as relative frequencies and were compared through Pearson’s chi-squared test or Fisher’s exact test, if appropriate. Relationships between variables were explored using Pearson’s linear correlation coefficient and Spearman’s nonparametric coefficient. Finally, we employed multiple regression and logistic regression models in order to evaluate the role and impact of predictive factors on the main endpoints of the study.

## 3 Results

### 3.1 Study subjects

We identified 86 consecutive patients who had been prescribed with mepolizumab between June 2017 and May 2018. We evaluated the presence of missing data, the compliance with the pre-specified inclusion and exclusion criteria and contacted them in order to sign the informed consent. Out of 86 patients, seven patients were missing crucial data at either T_0_, T_12_ or T_72_; two patients died from causes unrelated to SA; nine patients were lost to follow-up. Finally, two patients refused to participate in the study (Supplementary Figure S1). Therefore, we included in the final analysis 62 patients (40 women, 64.5%) with a mean age of 61.7 ± 12.6 years and a mean disease length of 34.3 ± 10.9 years. All of them signed a written informed consent. Our sample was mostly made up of never-smokers, OCS-dependent, atopic and hypereosinophilic patients with a high degree of comorbidities (43.6% of them had at least two ongoing concomitant conditions), as shown in Table 1.

**TABLE 1:** Baseline characteristics and comorbidities of the patients included in the study. Data are expressed as mean ± standard deviation or median (interquartile range), unless otherwise specified.

Demographics	
N	62
Age, years	61.7 ± 12.6
BMI	27.0 ± 5.0
Disease length, years	34.3 ± 10.9
Females, n (%)	41 (66)
Atopy, n (%)	51 (82)
Smoking history, n (%)	14 (22.6)
Comorbidities	
CRSwNP, n (%)	31 (50)
OSAS, n (%)	3 (0.4)
Anxiety, n (%)	2 (0.3)
GERD, n (%)	15 (24)
Bronchiectasis, n (%)	17 (27)
Hypertension, n (%)	21 (34)

Abbreviations: n, number; BMI, body mass index; CRSwNP, chronic rhinosinusitis with nasal polips; OSAS, obstructive sleep apnea syndrome; GERD, gastroesophageal reflux disease.

### 3.2 Effects of mepolizumab on clinical control

**FIGURE 1:**
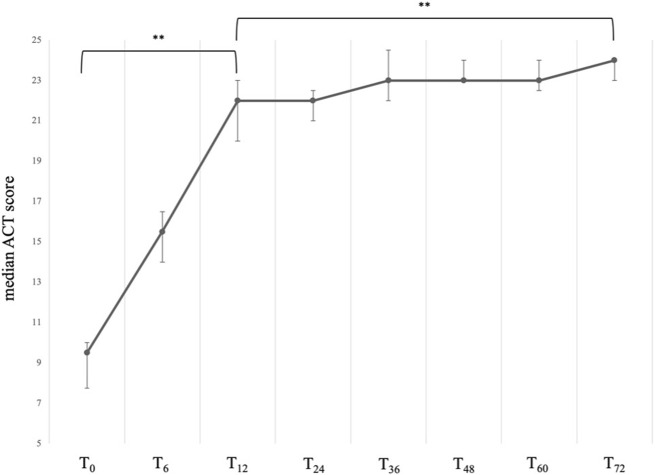
Variation of the ACT total score across timepoints. Data are presented as median (points) and interquartile range (bars). ** = variation is significant at *p* < 0.001. T_0_ = baseline. T_6_ = 6-month follow-up. T_12_ = 12-month follow-up. T_24_ = 24-month follow-up. T_36_ = 36-month follow-up. T_48_ = 48-month follow-up. T_60_ = 60-month follow-up. T_72_ = 72-month follow-up.

At baseline, our sample presented with a severely uncontrolled asthma, with a median ACT score of 9.50 (IQR:7.75; 10.00). After starting treatment with mepolizumab, patients showed a sustained and significant improvement in their quality of life (Figure 1), with the greatest improvements occurring within the first year of treatment. In particular, between T_0_ and T_12_, we observed an average increase in the ACT score of 12.5 points, thus reaching a median value of 22.00 (IQR:20.00; 23.00), *P* < 0.001. After the first year, a slower but positive trend towards full asthma control was observed, and at 72 months a median value of 24.00 (IQR: 23.00; 24.00) was reported, *P* < 0.001 vs. T_0_ and *P* < 0.001 vs. T_12_ (Table 2).

**TABLE 2:** Comparison between baseline, one-year and six-year follow-ups. Data are expressed as mean ± standard deviation or median (interquartile range), unless otherwise specified.

Variables	T_0_	T_12_	T_72_	T_12_ vs. T_0_	T_72_ vs. T_0_	T_72_ vs. T_12_
Number of patients	62	62	62	—	—	—
Markers of T2 high inflammation						
Eosinophils, cell/mm^3^	772.50 (635.75; 888.75)	89.80 (49.5; 109.2)	91.50 (57.25; 104.25)	**<0.001**	**<0.001**	0.349
FeNO, ppb	47.81 ± 10.1	39.26 ± 6.65	35.85 ± 7.30	**<0.001**	**<0.001**	**<0.001**
Criteria for clinical remission						
ACT score	9.50 (7.75; 10.00)	22.00 (20.00; 23.00)	24.00 (23.00; 24.00)	**<0.001**	**<0.001**	**<0.001**
Exacerbations per year, n	4.0 (4.0; 5.0)	1.0 (0; 1.0)	1.0 (0; 1.0)	**<0.001**	**<0.001**	**0.014**
Exacerbation-free patients, n (%)	0 (0)	27 (38,7)	29 (46,7)	**<0.001**	**<0.001**	0.063
Daily OCS dose, mg	25.00 (12.50; 25.00)	0 (0; 1.00)	0 (0; 0)	**<0.001**	**<0.001**	0.070
Lung function						
FEV_1_, L	1.21 ± 0.48	1.50 ± 0.43	1.51 ± 0.43	**<0.001**	**<0.001**	0.088
FEV1%, % predicted	49.92 ± 10.75	59.18 ± 8.77	62.31 ± 7.91	**<0.001**	**<0.001**	**<0.001**
FVC, L	2.46 ± 0.67	2.56 ± 0.65	2.61 ± 0.68	**<0.001**	**<0.001**	0.115
FVC%, % predicted	80.6 ± 13.9	84.1 ± 10.9	88.7 ± 10.0	**<0.001**	**<0.001**	**<0.001**
FEV_1_/FVC, %	60.00 (57.00; 63.25)	69.20 (67.08; 72.78)	69.60 (67.18; 71; 73)	**<0.001**	**<0.001**	0.443

Abbreviations: FeNO: fractional exhaled nitric oxide; ACT, asthma control test; OCS, oral corticosteroids; FEV_1_, forced expired volume in the 1^st^ second; FVC, forced vital capacity; N, number; L, liters; %, percentage. Bold values: result is significant at *p* < 0.05.

**FIGURE 2:**
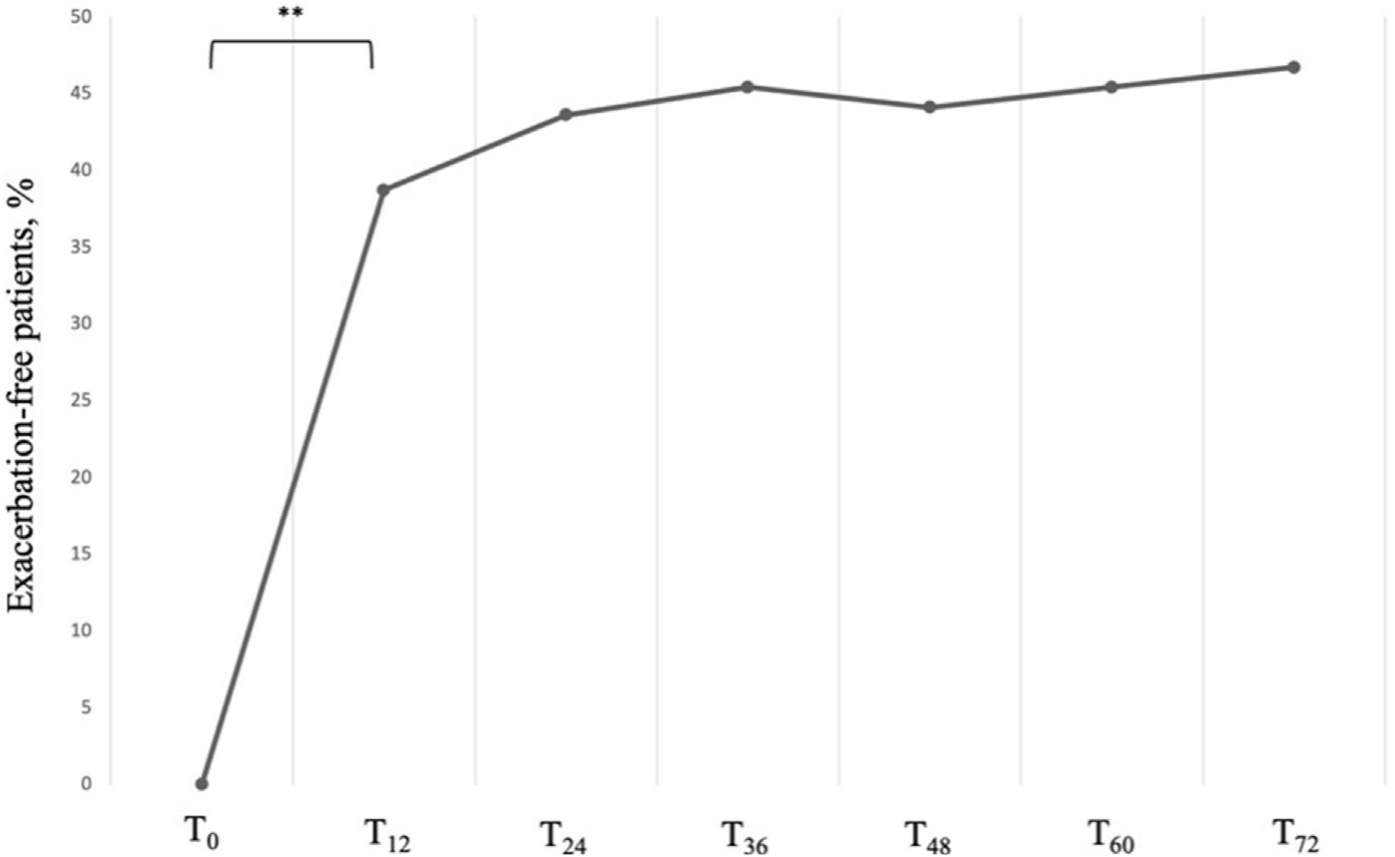
Proportion of exacerbation-free subjects across timepoints. Data are presented as cumulative percentages. ** = variation is significant at *p* < 0.001. T_0_ = baseline. T_12_ = 12-month follow-up. T_24_ = 24-month follow-up. T_36_ = 36-month follow-up. T_48_ = 48-month follow-up. T_60_ = 60-month follow-up. T_72_ = 72-month follow-up.

Accordingly, we observed a significant and sustained reduction in the AER, which dropped from a baseline value of 4.00 (IQR: 4.00; 5.00) per year to a much lower median value of 1.00 (IQR: 0.00; 1.00) per year at 12 months (*P* < 0.001). The control of the exacerbation rate was then maintained up to 72 months without any significant variation, with a growing number of exacerbation-free patients, which went from 0 at baseline to 29 (46,7%) at T_72_ (Figure 2).

**FIGURE 3:**
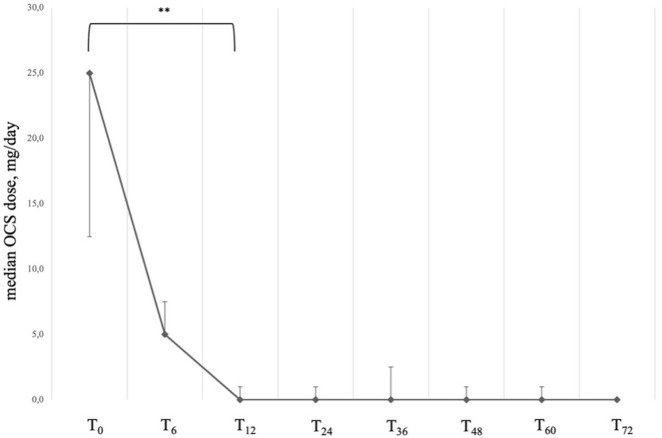
Variation of OCS daily dose across timepoints. Data are presented as median (points) and interquartile range (bars). ** = variation is significant at *p* < 0.001. T_0_ = baseline. T_6_ = 6-month follow-up. T_12_ = 12-month follow-up. T_24_ = 24-month follow-up. T_36_ = 36-month follow-up. T_48_ = 48-month follow-up. T_60_ = 60-month follow-up. T_72_ = 72-month follow-up.

At baseline, all patients included in our sample were taking systemic steroids on a daily basis (Table 2). After 12 months of treatment with Mepolizumab, the average daily dosage was found to be reduced from an average intake of 25.00 (IQR: 12.50; 25.00) mg/day to 0.00 (IQR: 0.00; 1.00) mg/day after 12 months (*P* < 0.001); after 72 months of treatment, the daily average intake of OCS remained stable, with the 85% of our sample reaching the complete elimination of OCS from stable therapy (Table 3; Figure 3). It is also noteworthy that the steroid-sparing effect occurred rapidly and was clinically significant, since 72.6% of our patients obtained a reduction ≥90% of the daily OCS dose within 12 months from baseline (Table 3).

**TABLE 3:** Extent of OCS reduction at 12 (T_12_), 36 (T_36_) and 72 (T_72_) months after treatment start with mepolizumab.

Extent of OCS dose reduction	T_12_	T_36_	T_72_
Any reduction, n (%)	61 (98.4)	62 (100)	62 (100)
≥90%, n (%)	45 (72.6)	50 (80.6)	53 (85.5)
≥75%, n (%)	53 (85.5)	53 (90.3)	56 (91.9)
≥50%, n (%)	60 (96.8)	62 (100)	62 (100)
≥25%, n (%)	61 (98.4)	62 (100)	62 (100)
No reduction, n (%)	1 (1.6)	0 (0)	0 (0)
Elimination, n (%)	45 (72.6)	50 (80.6)	53 (85.5)

### 3.3 Effect of mepolizumab on clinical remission

The application of the above-mentioned criteria to our sample allowed us to identify 18 patients (29.0%) who reached cCR at T_12_, and 27 (43.5%) at T_72_, while the patients who reached pCR were 38 (61.3%) at T_12_ and 53 (85.5%) at T_72_.

A comparison between patients who achieved cCR with those who did not was made in order to identify differences and potential predictors of outcome (Table 4). No difference was found in demographical data, lung function, biomarker expression and PRO at baseline; the only statistically significant difference, however, was found in the number of previous acute exacerbations at baseline, which was higher among non-cCR patients. We therefore investigated possible relationships between variables through univariate and multivariate logistic regressions. At the univariate analysis, sex category, atopy, BMI, CRSwNP, bronchiectasis, anxiety, baseline FEV_1_, baseline OCS dose and baseline ACT did not reach statistical significance while a higher number of exacerbations at baseline was associated to a lower probability of reaching cCR. After adjusting for sex, atopy, BMI, presence of CRSwNP, presence of bronchiectasis and mean OCS dose at baseline, the number of exacerbations at baseline was confirmed to be a negative predictor of cCR, with an adjusted Odds Ratio (OR) of 0.023 (95% confidence interval, CI: 0.002–0.227), *P* < 0.001.

**TABLE 4:** Comparison between patients who achieved clinical remission, either complete or partial, and patients who did not. Data are expressed as mean ± standard deviation or median (interquartile range), unless otherwise specified.

Variables	cCR, n = 27	Non-cCR, n = 35	*p*-value	pCR, n = 53	Non-pCR, n = 9	*p*-value
Age, years	64.3 ± 8.6	59.7 ± 14.8	0.131	62.8 ± 10.9	55.1 ± 19.6	0.283
Disease length, years	35.6 ± 8.5	33.2 ± 12.6	0.382	34.7 ± 10.1	31.9 ± 15.8	0.624
BMI	26.8 ± 4.6	27.0 ± 5.3	0.870	26.8 ± 4.8	27.7 ± 6.1	0.637
ACT baseline score	9.4 ± 2.3	8.8 ± 2.7	0.377	9.2 ± 2.5	8.6 ± 2.7	0.506
FEV_1_ baseline, L	1.15 ± 0.53	1.26 ± 0.44	0.409	1.18 ± 0.48	1.39 ± 0.48	0.220
FEV1% baseline, % predicted	50.5 ± 12.0	49.5 ± 9.9	0.721	79.5 ± 13.7	86.9 ± 14.7	0.154
Exacerbations/y baseline	4.00 (3.00; 4.00)	4.00 (4.00; 5.00)	**<0.001**	4.0 (4.0; 4.0)	5.0 (4.0; 5.0)	**0.005**
OCS mean dosage baseline, mg/day	25.00 (12.50; 25.00)	25.00 (12.50; 25.00)	0.084	25.0 (12.5; 25.0)	25.0 (12.5; 37.5)	0.280
Eosinophils baseline, cell/mm^3^	743 (601; 943)	790 (654; 881)	0.594	764.0 (613.5; 905.0)	792.0 (664.5; 896.5)	0.583
IgE baseline, kU/L	469 (312; 742)	321 (123; 698)	0.194	423.0 (192.0; 720.0)	369.0 (69.5; 749.5)	0.667
FeNO baseline, ppb	47 (39; 58)	48 (39; 56)	0.194	47.0 (38.0; 54.50)	52.0 (49.0; 59.0)	**0.030**
Males, n (%)	7 (35)	14 (40)	0.246	15 (28.3)	6 (66.7)	**0.034**
Smoking history, n (%)	8 (29.6)	6 (17.1)	0.195	11 (20.8)	3 (33.3)	0.327
Atopy, n (%)	24 (88.9)	27 (77.1)	0.321	45 (84.9)	6 (66.7)	0.191
CRSwNP, n (%)	12 (44.4)	19 (54.3)	0.442	24 (45.3)	7 (77.8)	0.073
Bronchiectasis, n (%)	7 (25.9)	10 (28.6)	0.817	15 (28.3)	2 (22.2)	0.529
OSAS, n (%)	0 (0)	3 (0.09)	0.250	3 (5.7)	0 (0)	0.619

Abbreviations: BMI, body mass index; ACT, asthma control test; FEV1, forced expired volume in the 1^st^ second; OCS, oral corticosteroids; IgE, immunoglobulin E; FeNO, Fractional exhaled nitric oxide; CRSwNP, chronic rhinosinusitis with nasal polyps; OSAS, obstructive sleep apnea syndrome; N, number. L, liters; %, percentage. Bold values: result is significant at *p* < 0.05.

The comparison between pCR and non-pCR patients was also performed (Table 4). Here, a significant difference in baseline exacerbation rate and FeNO values was found. At the univariate analysis, sex category, FeNO and baseline exacerbation rate were found to be independent predictors of pCR. After adjusting for sex, atopy, BMI, presence of CRSwNP, presence of bronchiectasis and mean OCS dose at baseline, FeNO at baseline and the exacerbation rate at baseline were confirmed to be negative predictors of pCR, with an adjusted Odds Ratio (OR) of 0.855 (95% confidence interval, CI: 0.748–0.977), *P* = 0.020, and of 0.039 (95% confidence interval, CI: 0.002–0.597), *P* = 0.020, respectively.

### 3.4 Effects of mepolizumab on lung function and inflammatory biomarkers

**FIGURE 4:**
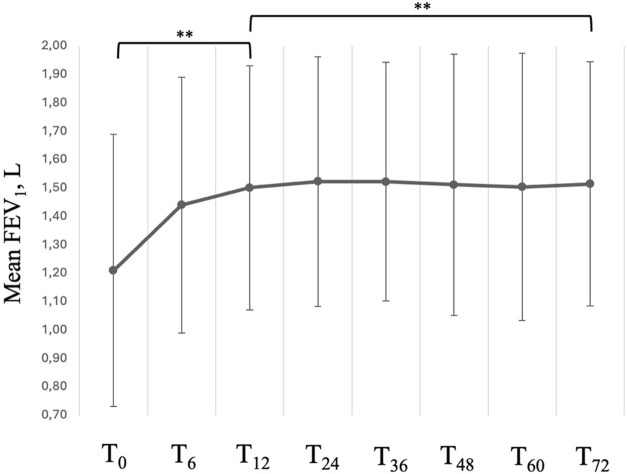
Variation in FEV_1_ across timepoints. Data are presented as mean (points) and standard deviation (bars). ** = variation is significant at *p* < 0.001. T_0_ = baseline. T_6_ = 6-month follow-up. T_12_ = 12-month follow-up. T_24_ = 24-month follow-up. T_36_ = 36-month follow-up. T_48_ = 48-month follow-up. T_60_ = 60-month follow-up. T_72_ = 72-month follow-up.

FEV_1_ increased from a mean value of 1.21 ± 0.48 L to 1.50 ± 0.43 L already at 12 months after treatment start (*P* < 0.001) with a mean variation (∆) of 300 mL from baseline (Figure 4); the magnitude of such variation was then maintained throughout the entire observational period (mean value of 1.51 ± 0.43 after 72 months of treatment, *p* = 0.088 vs. T_12_). When considering the variations in FEV_1_%, we observed a likewise statistically significant and rapid increase from a mean value of 49.92% ± 10.75% at baseline to 59.18% ± 8.77% at T_12_ (*P* < 0.001). Between T_12_ and T_72_, we observed a slow, but constant tend towards improvement, with a mean value of 62.31% ± 7.91% at T_72_ (*P* < 0.001 vs. T_12_). We observed similar trends of improvement also in FVC and FEV_1_/FVC ratio (Table 2).

Our patients underwent, as part of our center’s protocol for the assessment of severe asthma, repeated measurements of the exhaled fraction of nitric oxide (FeNO) and BEC (Table 2). At baseline, FeNO was found to be elevated in most patients, with an average value of 47.81 ± 10.1 ppb, thus reflecting a severely dysregulated T2-inflammation pathway. At T_12_, the mean FeNO value was reduced to 39.26 ± 6.65 ppb (*p* < 0.001); the trend towards normalization slowed down after 12 months but kept towards reduction reaching an average value of 35.85 ± 7.30 ppb at T_72_, (*p* < 0.001 vs. T_12_), as shown in Supplementary Figure S2.

The drug’s action against IL-5 also resulted in a drastic reduction in the number of circulating eosinophils, which dropped from a median pretreatment value of 772.50 (IQR:635.75; 888.75) cell/mm^3^ to 89.80 (IQR:49.5; 109.2) cell/mm^3^ at T_12_. The extent of such reduction was then maintained for the following years, with no further significant variations between timepoints (Supplementary Figure S2).

### 3.5 Mepolizumab-related ADRs

At the first administration of mepolizumab, patients remained under observation for at least 2 h before discharge. The following injections were self-administered at home. At the end of the observational timeframe, no hypersensitivity nor anaphylactic reaction, as well as no severe ADRs were reported. Under no circumstance did any patient discontinue the drug due to ADRs.

At baseline, within 24 h from the drug administration, 13 (21.0%) patients reported headache, six (9.7%) patients reported injection site reactions and two (3.2%) reported a slight temperature arousal (between 37.1°C and 37.8°C). Three patients (4.8%) reported two contemporary ADRs, while one (1.6%) reported more than two contemporary ADRs. During the following 6 years, 25 (40.3%) patients reported at least one episode of headache within 24 h from a scheduled drug administration, 12 (19.4%) reported at least one injection site reaction, and 5 (8.0%) reported at least one episode of lower back pain after an injection. Ten patients reported more than one occurrence of the same ADR; in particular: two patients reported at least two episodes of lower back pain, five patients reported at least two episodes of headache, and three patients reported at least two injection site reactions. Finally, nine patients (14.5%) reported at least two different ADRs during the observational period. All the reported ADRs were deemed to be mild in intensity, with no associated hospitalization or sick leaves, and had an average duration of 18.0 ± 12.0 h.

None of the reported ADR led to withdrawal from therapy with mepolizumab. Finally, the most commonly prescribed drugs in order to achieve complete remission of the symptomatology were non-steroid anti-inflammatory drugs (NSAIDs).

## 4 Discussion

In our study we have shown that mepolizumab is able to significantly improve asthma control, reduce AER and eliminate or decrease the maintenance OCS dose within the first year of treatment and then maintain such achievements for the following 5 years.

To our knowledge, this is the first study that involved patients followed without interruption for six whole years after treatment start. Some previous studies investigated the long-term effects of mepolizumab treatment in SEA patients (Khatri et al., 2019; Khurana et al., 2019; Domingo Ribas et al., 2021; Moore et al., 2022; Pilette et al., 2022; Fyles et al., 2023), covering a median timespan of about 3 years. One of such studies (Fyles et al., 2023) included both patients treated with mepolizumab and benralizumab (an anti-IL-5 receptor drug) and presented most of the results for the overall population, thus making it difficult to extrapolate the data regarding the sole mepolizumab-treated patients. Finally, only one retrospective single-center study (Strauss et al., 2023) which included a total of 67 patients enrolled patients treated from a minimum of 6 months up to a maximum of six and half years. This large variability in treatment time, however, diluted the possibility of inferring the persistence of the treatment-related effects over the years. Our study therefore confirms the results of other research teams, which underscored a huge impact of mepolizumab on asthma control, AER and reduction of OCS doses, and adds precious information on the stability of both clinical and functional parameters of SEA patients treated with mepolizumab at 6 years.

Before the introduction of biological drugs, OCS have been extensively used to reduce symptoms and prevent exacerbations, thus guaranteeing at least an apparently better asthma control (Ortega et al., 2019). However, OCS are burdened by several side effects, which alter the homeostasis of the patients and eventually lead to increased morbidity and mortality, and therefore their use should be avoided (Bourdin et al., 2017; Price et al., 2018; Lee et al., 2019; Lommatzsch et al., 2023). In this context, the effects of mepolizumab as a steroid-sparing drug have been reported both in RCTs and real-life studies. In particular, the prospective multicenter REALITI-A study (Pilette et al., 2022) involved a cohort of 822 patients, of whom 319 were taking maintenance OCS at enrolment. After 52 weeks from index, 43% of patients had managed to eliminate the maintenance OCS, while another 21% of patients experienced a reduction of the OCS maintenance dose between 50% and 99% of the baseline value. The promising results of the REALITI-A protocol, however, were affected by the outbreak of the COVID-19 pandemics, and only the 82% of the initial sample could complete the observational period. Nonetheless, a meaningful impact of mepolizumab on steroid maintenance therapy was demonstrated, although the study did not provide any information about the maintaining of such effect over time. In this context, the Spanish multicenter, retrospective cohort study “REDES” (Domingo Ribas et al., 2021) studied the records of over three hundred patients with OCS-dependent SEA and corroborated the evidence that patients treated with mepolizumab experienced a dramatic improvement both in the annual exacerbation rate and in the daily OCS maintenance dose. Our results seem to be in line with the ones reported in both REALITI-A and REDES trial, with comparable rates of reduction in maintenance OCS doses and AER, as well as comparable proportions of exacerbation-free patients at 6 years. Thus, our findings seem to corroborate the idea that the treatment-related benefits might be sustained for a much longer time.

We observed a rapid increase in the FEV_1_ values of our patients, with a mean gain of 300 mL over the first year of treatment; such increase was stabilized after 1 year, even if a slight but constant tendency towards improvement was noticed in the following years. However, it must be noticed that our patients were particularly impaired in their lung function at baseline when compared to the cohorts of other studies, probably as a consequence of a higher mean age and a longer story of uncontrolled asthma. Therefore, the mean FEV_1_% did not reach the threshold for normality, being it under the 80% even after 72 months. This may reflect the remodeling processes that take place among asthmatic patients who do not reach control of their condition, which can lead to small airway dysfunction (SAD) and its clinical surrogate manifestation, namely lung hyperinflation (van der Meer et al., 2019). Previous evidence has shown that biologics are able to reduce the extent of lung hyperinflation and thus improve the patient’s functional status (Maniscalco et al., 2024). However, while there is no evidence of the efficacy of mepolizumab on the reduction of the residual volume, Strauss et al. (2023) indicated that mepolizumab was able to improve the airflow in the small airways, with a marked improvement in the forced expiratory flow at the 25%–75% of the pulmonary volume (FEF_25-75_), a spirometric parameter that to an extent reflects SAD (Siroux et al., 2016). Nonetheless, the fact that we recorded a slight, but constant improvement in FEV_1_ values over the years hints at the possibility of a slight, but constant action of mepolizumab on the SAD and its clinical and functional consequences, reflected by a sheer improvement of the quality of life and a reduction in the AER. Conversely, it could be also hypothesized that an earlier treatment start with mepolizumab might have an even more impactful effect on lung function thanks to an earlier action against airway remodeling; however, such hypothesis should be verified with *ad hoc* designed studies that should also investigate, whenever possible, the permanence of such effects after treatment discontinuation.

As previously reported in other studies (Khatri et al., 2019; Khurana et al., 2019; Domingo Ribas et al., 2021; Fyles et al., 2023), mepolizumab affected the well-established biomarkers of T2-high inflammation, inducing a slight but significant reduction in FeNO values, as well as a marked, sevenfold fall in the eosinophilic count within the first year of treatment; such a result was maintained throughout the entire observational timeframe. The action of mepolizumab on eosinophils seems to be more regulatory rather than suppressory, as shown in a recent study (Vultaggio et al., 2023); therefore, our patients experienced a stabilization of the BEC rather than its complete elimination. The implications of such piece of evidence are yet to be determined, but the return to a condition of homeostasis in the complex biology of eosinophils might have a long-lasting positive impact on the whole organism (Candia et al., 2024).

Both the REALITI-A (Pilette et al., 2022) and the REDES (Domingo Ribas et al., 2021) studies offered the opportunity to identify predictive factors for the so-called “super response” to therapy. In particular, a *post hoc* analysis of the REDES study (Pavord et al., 2023) reported a 30% proportion of cCR defined as the contemporary presence of four criteria, including a reversion to a FEV1% ≥ 80%. In our experience, the percentage of patients reaching cCR at 6 years was higher (47%); moreover, it is noticeable that 18 patients (29.0%) had already reached cCR at 12 months, thus suggesting that a prolonged treatment with mepolizumab might result in higher cCR rates.

The same *post hoc* analysis (Pavord et al., 2023) identified several outcome predictors, namely a higher baseline BEC, a better asthma control in terms of ACT score, a lower exacerbation rate and a lower mean maintenance OCS dose at baseline. Accordingly, in our study, we explored both continuous and categorial variables in order to identify predictors of CR. Baseline characteristics such as sex, BMI and comorbidities, in particular chronic rhinosinusitis with nasal polyps and bronchiectasis did not reach statistical significance. Our results might be related to the high BEC at baseline and to the overall impaired lung function found in our sample; however, the exacerbation rate at baseline seems to negatively affect the possibility of reaching both cCR and pCR. This finding might depend on the degree of severity of the airway remodeling (Krings et al., 2021), thus suggesting that clinicians should not wait long before initiating a treatment with mepolizumab, whenever SEA is diagnosed.

Interestingly enough, 85.5% of our sample reached a pCR, which mandatorily included the elimination of chronic treatment with OCS. Overall, whether we consider cCR or pCR, we demonstrated that mepolizumab has an impactful and sustained effect on steroid reduction. In particular, within a year from treatment start, almost 75% of our patients had ceased to take OCS regularly. This proportion reached the outstanding percentage of 85% at 6 years. This evidence contributes to indicate that mepolizumab is effective in limiting of the OCS-related side effects, which worsen outcomes and impair the quality of life of SEA patients (Price et al., 2018). There is a significant difference between the REDES criteria for cCR and the ones we employed in this study, which were based on a recent Delphi consensus (Canonica et al., 2023). In particular, the main discordance lies in the definition of lung function stability: while in the REDES study a FEV1% ≥ 80% was considered as a criterion for cCR, we rather considered null or positive variations in FEV1% between timepoints to define the stability of lung function, given the premises discussed above.

Finally, mepolizumab proved to be effective, well-tolerated and generally safe after a long-term continuous treatment. In fact, no severe reaction was observed in our sample, in line with what was observed in the REDES study (Domingo Ribas et al., 2021). We reported a higher prevalence of mild ADRs, with 21 patients (33.9%) having at least one ADR after the first injection. This increased prevalence of ADRs among our patients might partly be related to our sample size and its characteristics, such as the higher age and the burdening presence of both respiratory and non-respiratory comorbidities. It is indeed known that age and multimorbidity are both established risk factors for ADRs among older adults due to multifactorial changes in drug metabolism and the increased risk of pharmacological interactions (Zazzara et al., 2021). Accordingly, in comparison to the REDES cohort, our sample was older (mean age 61.7 ± 12.6 vs. 56.6 ± 12.5). Nonetheless, the overall profile of safety and tolerability was demonstrated to be satisfactory, suggesting that mepolizumab can be used also in older adults without peculiar concerns.

This study has several strengths, since, to our knowledge, it is the first one that was specifically designed to investigate the long-term effects of mepolizumab in a homogeneous cohort of SEA patients. All patients were in fact evaluated by the same team of physicians, thus improving the standardization and comparability of data. Patients were carefully screened for inclusion so as to reduce at the bare minimum any potential confounding factor.

However, some limitations of our protocol must be addressed, since they may affect the overall reliability of our results. In particular, the retrospective design and the very old age of our population might have concealed some of the effects of mepolizumab. In fact, as discussed before, it must be considered that lung function tends towards a natural decline with aging, and that a long history of disease might have had an impact of airway remodeling. Also, the absence of a control group has an impact on the power of our conclusions. While on one hand the single-center recruitment guaranteed homogeneity, on the other hand it has an inevitable impact on the generalizability of our results, which derive from patients who lived and worked in a rather limited area of Southern Italy (Campania). We also believe that the influx of SARS-CoV2 cannot be excluded, and that we could not ascertain its effects on the patients included in our study without any reasonable doubt.

## 5 Conclusion

Our study pointed out that mepolizumab is effective and well-tolerated after a six-year course of continuous treatment in patients with SEA.

In fact, quality of life, AER and maintenance OCS dose were improved after 1 year of treatment and such improvement persisted for the following 5 years in the vast majority of our sample. Mepolizumab proved also effective in ameliorating FEV_1_, despite the highly impaired lung function at baseline, and reduced both BEC and FeNO values. We observed that a significant proportion of our study population reached the criteria for cCR already at T_12_, and that this proportion increased steadily over the years up to the 46.8% at T_72_, thus providing further insights on the sustained efficacy of mepolizumab and evidence supporting the long-term use of the drug thanks to its disease-modifying action. Finally, mepolizumab was found to be safe, with no severe ADR reported. Despite some limitations, this is the first study that investigated the effectiveness and tolerability of mepolizumab in a cohort of Italian patients after such a long-lasting time. Further studies, possibly multicenter and prospective, will be needed in order to corroborate our findings and to strengthen our knowledge of mepolizumab and its beneficial and multidimensional effects on patients suffering from SEA.

## Data Availability

The raw data supporting the conclusions of this article will be made available by the authors, upon reasonable request to the corresponding author.

## Supplementary material

SUPPLEMENTAL FIGURE S1 Flowchart of patient enrolment and selection.

SUPPLEMENTAL FIGURE S2 Effect of mepolizumab on T2-high inflammation biomarkers, namely FeNO **(A)** and blood Eosinophils **(B)**. Data are expressed as mean and standard deviation (error bars) or median and interquartile range (error bars) as appropriate. Abbreviations: FeNO, fractional exhaled nitric oxide. Eos, eosinophils.
