# Supplementary material for: Long-term effects of mepolizumab in patients with severe eosinophilic asthma: a 6-year real-life experience
Source: Front Pharmacol. 2024 Aug 8;15:1449220. doi: 10.3389/fphar.2024.1449220 (PMC11338755; doi:10.3389/fphar.2024.1449220)
Supplement: Supplementary file 1 [file Table1.docx]

Supplemental Material

**Long-term effects of mepolizumab in patients with severe eosinophilic asthma: a 6-year real-life experience**

Anna Agnese Stanziola^1^, Claudio Candia^1^, Gerardo Nazzaro^1^, Antonio Caso^1^, Claudia Merola^2^, Mauro Maniscalco^1,2^

^1^ Department of Clinical Medicine and Surgery, University of Naples “Federico II”, 80131 Naples, Italy;

^2^ Istituti Clinici Scientifici Maugeri IRCCS, Pulmonary Rehabilitation Unit of Telese Terme, 82037 Telese Terme, Italy.

**Supplemental Table 1** – Mepolizumab prescription criteria for severe asthma, according to the Italian Drug Regulatory Agency (Agenzia Italiana del Farmaco, AIFA). In order to proceed with the prescription, the patient must comply with criterion A and at least one between B or C. Abbreviations: BEC, blood eosinophil count.

| 1. Patients ≥ 6 years of age with a BEC > 150 cells/mm^3^ in absence of any steroid treatment at the first prescription and evidence of at least one BEC > 300 cell/mm^3^ within the previous 12 months. |
| --- |
| 1. One of the following conditions: 2. Patients ≥ 12 years old: At least 2 acute exacerbations despite maximal inhalatory therapy which required treatment with systemic steroids or hospitalization within the previous 12 months (GINA Steps 4-5). 3. Patients between 6-11 years old: At least 2 acute exacerbations despite the maximally tolerated inhalatory therapy which required treatment with systemic steroids or hospitalization within the previous 12 months. |
| 1. Patients ≥ 18 years of age who received a continuous treatment with systemic steroids in addition to maximal inhaled therapy for at least 6 months during the last year. |
