# Supplementary figures and images for: Long-term effects of mepolizumab in patients with severe eosinophilic asthma: a 6-year real-life experience
Source: Front Pharmacol. 2024 Aug 8;15:1449220. doi: 10.3389/fphar.2024.1449220 (PMC11338755; doi:10.3389/fphar.2024.1449220)

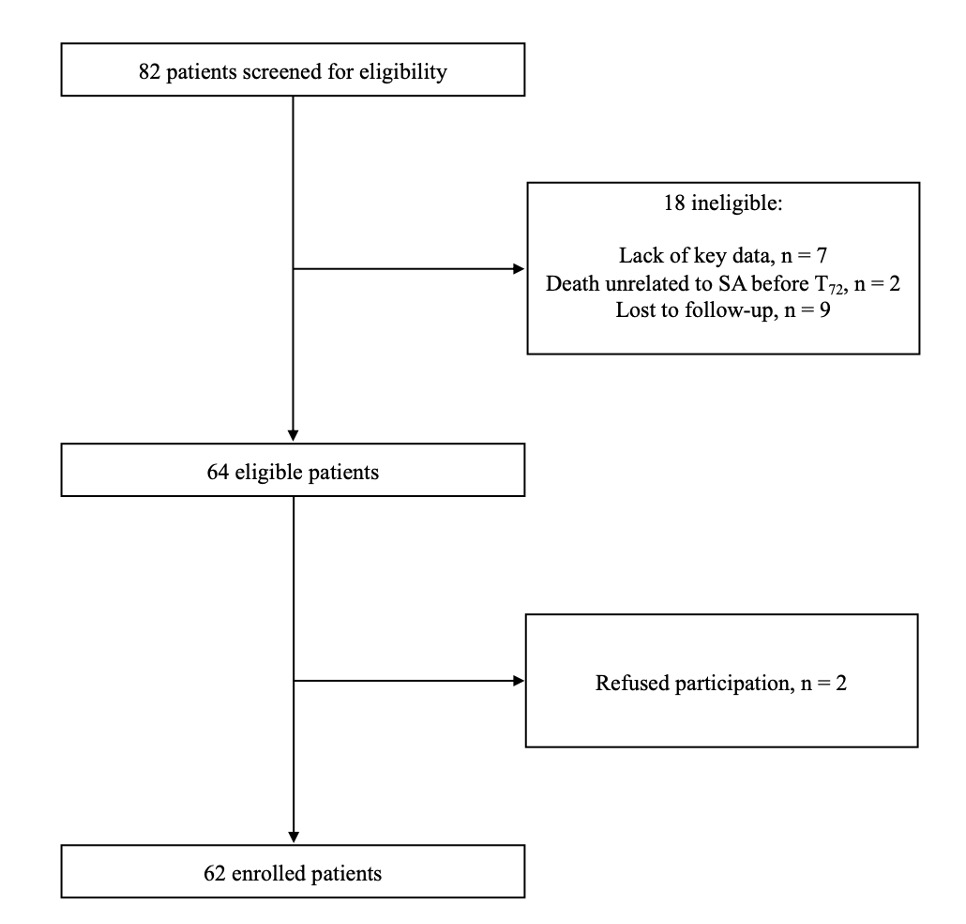

Supplement: Supplementary file 2 [file Image1.JPEG]

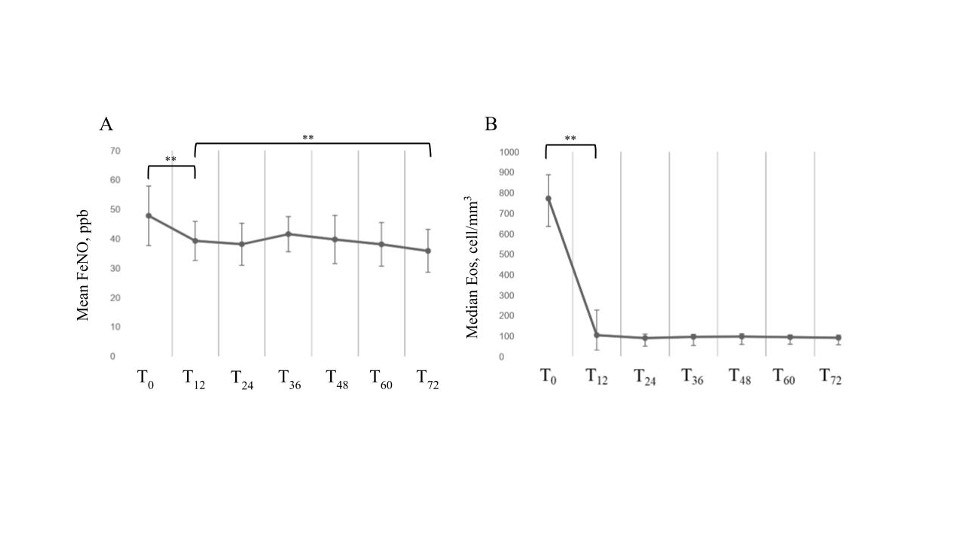

Supplement: Supplementary file 3 [file Image2.JPEG]
